# Effect of Heat Stress on Yield, Monoterpene Content and Antibacterial Activity of Essential Oils of *Mentha* x *piperita* var. Mitcham and *Mentha arvensis* var. *piperascens*

**DOI:** 10.3390/molecules23081903

**Published:** 2018-07-30

**Authors:** Milad Heydari, Anna Zanfardino, Alireza Taleei, Ali Akbar Shahnejat Bushehri, Javad Hadian, Viviana Maresca, Sergio Sorbo, Michela Di Napoli, Mario Varcamonti, Adriana Basile, Daniela Rigano

**Affiliations:** 1 Department of Agronomy and Plant Breeding, Collage of Agriculture and Natural Resources, University of Tehran, P.O. Box 31787-316 Karaj, Iran; milad.heydari@ut.ac.ir (M.H.); ashah@ut.ac.ir (A.A.S.B.); 2 Department of Biology—University of Naples “Federico II”, 80126 Naples, Italy; anna.zanfardino@unina.it (A.Z.); viviana.maresca@unina.it (V.M.); michela.dinapoli@unina.it (M.D.N.); mario.varcamonti@unina.it (M.V.); 3 Medicinal Plants and Drug Research Institute, ShahidBeheshti University, G.C. Tehran 11369, Iran; j_hadian@sbu.ac.ir; 4 C.e.S.M.A. University of Naples “Federico II”, 80126 Naples, Italy; sersorbo@unina.it; 5 Department of Pharmacy, School of Medicine and Surgery, University of Naples Federico II, 80126 Naples, Italy; drigano@unina.it

**Keywords:** *Mentha* x *piperita*, *Mentha arvensis*, essential oils, heat stress, antibacterial activity, monoterpenes

## Abstract

Heat stress affects the yield of medicinal plants and can reduce biomass and/or metabolite production. In order to evaluate the effect of heat-induced stress on the essential oil production in *Mentha* x *piperita* L. var. Mitcham (Mitcham mint) and *Mentha arvensis* var. *piperascens* Malinv. ex L. H. Bailey (Japanese mint), we studied the chemical composition of the oils of the two mint species under different heat shock stresses in growth chambers. The antibacterial activity of the essential oils was also evaluated; microscopic observation (fluorescence and electron transmission) was used to assess the effect of the tested samples on bacterial growth. The results obtained shed light on the mint essential oils composition and biological activity in relation to heat stress.

## 1. Introduction

*Mentha* x *piperita* L. and *Mentha arvensis* L. are perennial plants belonging to Lamiaceae family, originating from Europe but spread around the world and cultivated in many different climates. *M. piperita* (2n = 72) is a hexaploidy medicinal plant considered to be a sterile plant hybrid of *M. spicata* L. (Native Spearmint, 2n = 48 or 36) and *M. aquatica* L. (Water Mint, 2n = 96) [1]. *M. arvensis*, popularly known as wild mint or corn mint, has its unique importance among mint family due to its high content of menthol [2]. These *Mentha* species are two important medicinal plants due to high consumption in the world and for the size of the area cultivated for essential oil production [3]. They are reported in literature to be useful for the treatment of intestinal colic, spasms of the bile duct, dyspepsia, biliary, gallbladder and gastrointestinal (GI) tract disorders, gastritis, flatulence and enteritis [4].

Many medical properties of *Mentha* species are ascribed to their essential oil, that has been defined by monographs in several compendia, including the pharmacopeia from the United States, Great Britain, Japan and Europe, as well as many National Formulary collections and The Food Chemicals Codex. The essential oils generally include menthol, menthone, isomenthone, 1,8-cineole (eucalyptol), menthyl acetate, menthofuran, limonene, β-myrcene, β-caryophyllene, pulegone and carvone [5]. In particular, the most familiar secondary metabolite is menthol, whose consumption is more than 7000 ton/year (more than 300 million dollars) [3]. This metabolite is produced in peltate glandular trichomes located on the aerial parts of the mint plants [3,6].

In *M. piperita*, there are two major pathways to produce monoterpenes and sesquiterpenes, that are the most important secondary metabolite in the volatile oils. The MEP pathway produces monoterpenes from pyruvate, while the MVA pathway produces sesquiterpenes from Acetyl Co. A [5]. During the MEP pathway, pyruvate is converted to limonene in plastids. After the transfer to endoplasmic reticulum, limonene is converted to isopiperitenol, that in mitochondria changes to isopiperitenone. After that, isopiperitenone is released in the cytoplasm and converts to pulegone, that in cytoplasm produces menthol while in endoplasmic reticulum produces menthofuran [5]. Other components and their percentages are summarized in McKay et al. (2006) [4]. The metabolic pathway responsible for *M. arvensis* essential oil biosynthesis is well described in Tiwari et al. (2016) [7].

Although medicinal plants have considerable adaptability to a large spectrum of conditions, in certain environmental conditions they can reduce their potential to produce biomass and metabolites. Different abiotic stresses affect productivity and growth of plants such as temperature, content of salt, drought, anaerobic and flooding conditions [8]. The chemical composition of mint essential oils was showed to be affected by many factors, such as cultivar and environmental conditions, humidity, the nutrient in soil, temperature and biotic and/or abiotic stress [9]. One of the factors that can affect essential oil production is heat stress. Heat (or cold) stress can have murderous effects on plants [10,11]. The greenhouse effect increases the temperature in the world and changes the climates, therefore heat is becoming a major abiotic stress and has been recognized as an agrarian effect in various semi-arid and arid regions. High temperature creates a series of physiological and biochemical modifications in plants, which affect plant growth and development and can lead to acute reduction in economic yields [12,13]. Unfortunately, there is not enough information about the possible effect of heat stress on metabolite production and biological activities of medicinal plants.

Based on the above, the objective of this research was to investigate the changes in the quality, quantity and antibacterial activity of essential oils of *Mentha* x *piperita* L. var. Mitcham (Mitcham mint) and *Mentha arvensis* var. *piperascens* Malinv. ex L. H. Bailey (Japanese mint) under heat stress conditions. To investigate the effect of heat stress on essential oil composition in the two *Mentha* species, three different temperature conditions in growth chambers were used [14,15]. Microscopic observation (fluorescence and electron transmission) was used to assess the effect of the tested samples on bacterial growth.

## 2. Results

**Figure 1:**
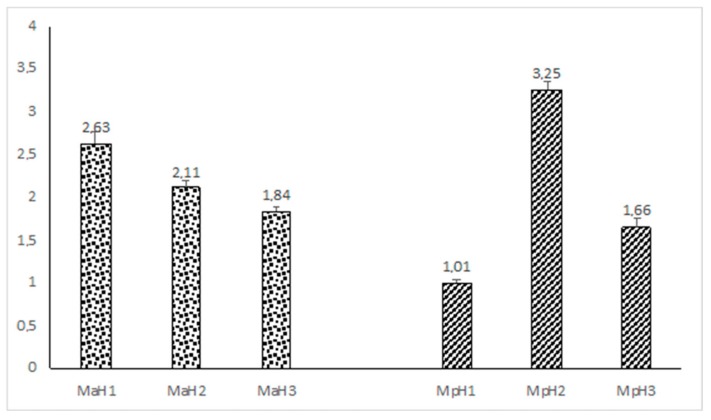
Yields (% *w*/*w* based on dry weight) of essential oils of the two mint species (Mp = *M. piperita*, Ma = *M. arvensis*, H1, H2 and H3 are the three tested temperatures). Each value is the mean ± SD of three independent measurements.

The essential oils from the six samples of mint leaves (Mp = *M. piperita*, Ma = *M. arvensis*, H1, H2 and H3 are the three tested temperatures, see Section 4.2) were found to be a yellow liquid with a pleasant smell. The average yields (% *w*/*w* based on dry weight) of extracted essential oils are shown in Figure 1.

The relative concentrations of the volatile components identified in the essential oils of the six samples are presented in Table 1, according to their retention indices Ki on a HP-5 MS column. A total of 26 compounds were identified in the oils. On the whole, qualitative profiles of all essential oils were characterized by a greater amount of oxygenated monoterpenes (88.3–94.8%) and particularly five compounds were the most representative: menthone (7.6–15.9%), menthofuran (7.8–35.0%), menthol (5.6–56.6%), pulegone (5.6–28.1%) and menthyl acetate (2.1–15.4%). As regards the single components, the peppermint oil was characterized by higher amounts of menthol, that for the sample MpH1 accounted for more than the half of the total oil (56.6%) but was also abundant in MpH2 and MpH3 (38.5% and 27.5%, respectively). For *M. arvensis* oil, the compound present in higher percentages was instead menthofuran, accounting for 35.0% of the total oil in MaH1, 25.4% in MaH2 and 34.0% in MaH3.

Other components present in minor concentrations in the six essential oils were monoterpene hydrocarbons (3.6–6.7%), sesquiterpene hydrocarbons (0.6–6.4%), oxygenated sesquiterpenes (0.2–0.3%) and others (0.8–2.3%).

**Figure 2:**
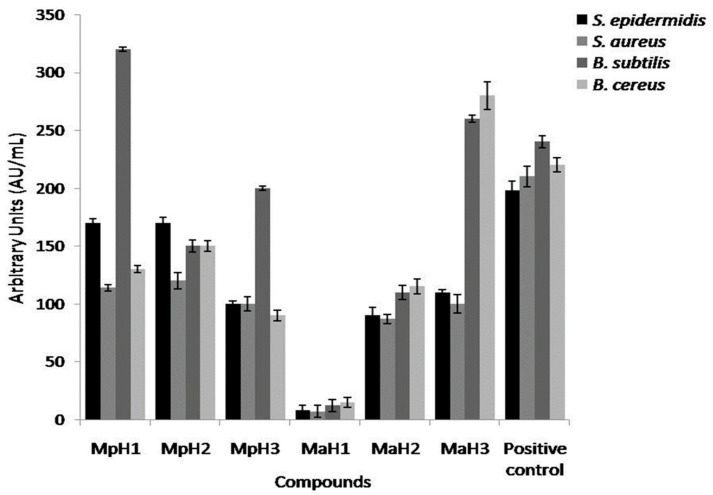
Inhibition halo of oils from *Mentha* x *piperita* and *Mentha arvensis* against *Bacillus subtilis*, *Bacillus cereus*, *Staphylococcus aureus* and *Staphylococcus epidermidis* strains. The inhibition halos are expressed in AU/mL (see methods). Positive control is Ampicillin. Values are expressed as average of three different experiments; standard deviations were always less than 10%.

In order to characterize the antimicrobial activity of the oils extracted from the two different mint species, we have carried out *Kirby* and *Bauer* assays [16]. The normalization of oils inhibition halo is shown in Figure 2: as we can see, the oils were active against the four bacterial strains used for the experiments and they were particularly active against the bacilli. Generally, essential oils from *M. piperita* showed a higher antimicrobial activity than the others, while in the case of the oils extracted from *M. arvensis* the effect of temperature was essential to increase their activity against the tested bacteria.

**Figure 3:**
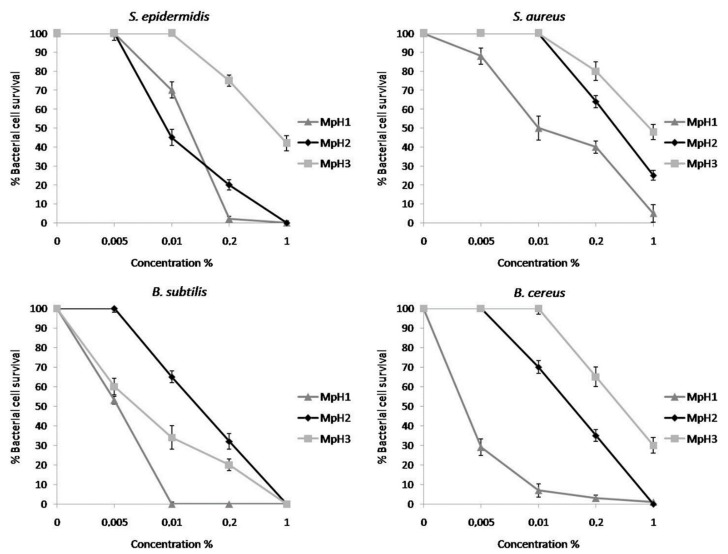
Antimicrobial activity of essential oils from *M. piperita* at different concentrations (0.005-0.01-0.2-1% *v*/*v*) valuated by colony count assay, after 2 h of incubation, against *Staphylococcus epidermidis, Staphylococcus aureus, Bacillus subtilis and Bacillus cereus strains*. The % of bacterial survival is represented on the *y* axis. The assays were performed in three independent experiments.

Successively we analysed separately, realizing dose-response curves, the oils extracted from *M. piperita*, using various % *v*/*v* as indicated in the methods. As shown in the Figure 3, MpH1 was the most active on all the bacterial strains used: in particular, at the concentration of 1%, it killed all the bacteria. Besides, increasing the temperature of the heat shock the antimicrobial activity seems to decrease, in all cases except for *S. aureus*. All the oils were more active on the bacilli and slightly less on *S. aureus* and *S. epidermidis*.

**Figure 4:**
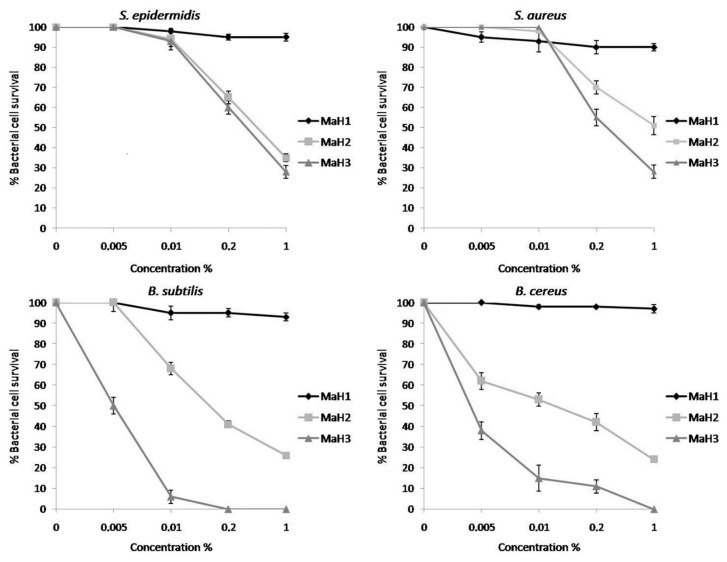
Antimicrobial activity of essential oils from *M. arvensis* at different concentrations (0.005-0.01-0.2-1% *v*/*v*) valuated by colony count assay, after 2 h of incubation, against *Staphylococcus epidermidis*, *Staphylococcus aureus*, *Bacillus subtilis* and *Bacillus cereus* strains. The % of bacterial survival is represented on the y axis. The assays were performed in three independent experiments.

The same dose-response curves were performed for *M. arvensis* oils. As shown in Figure 4, these oils had a lower antimicrobial activity compared to the oils extracted from *M. piperita*. The effect of heat shock stress seems to be necessary to obtain a better antimicrobial activity, which is the highest in the MaH3 oil for all strains analysed. Only on the bacilli and only for MaH3 to 1% of concentration *v*/*v* there is a complete death of the bacteria.

We were also able to estimate the Minimal inhibitory concentration (MIC). From the results shown in the Table 2 is clear that the MpH1 oil is certainly the most active oil even at low concentrations but in general all the of oils from *M. piperita*, possess a stronger antimicrobial activity. Furthermore, it is evident that all the tested oils have a greater activity on the bacilli (*Bacillus subtilis* and *Bacillus cereus*) rather than on the cocci (*Staphylococcus aureus* and *Staphylococcus epidermidis*).

**Figure 5:**
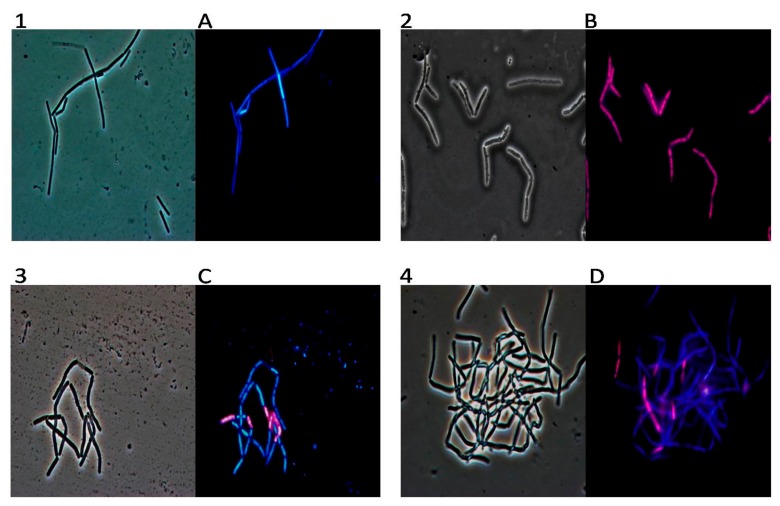
The panel shows *Bacillus cereus* bacterial cells observed in optical microscopy (numbers) and in fluorescence microscopy (letters). Untreated bacterial cells (**1**,**A**); cells treated with oil from MpH1 (**2**,**B**), cells treated with oil from MpH2 (**3**,**C**) and finally with oil from MpH3 (**4**,**D**).

**Figure 6:**
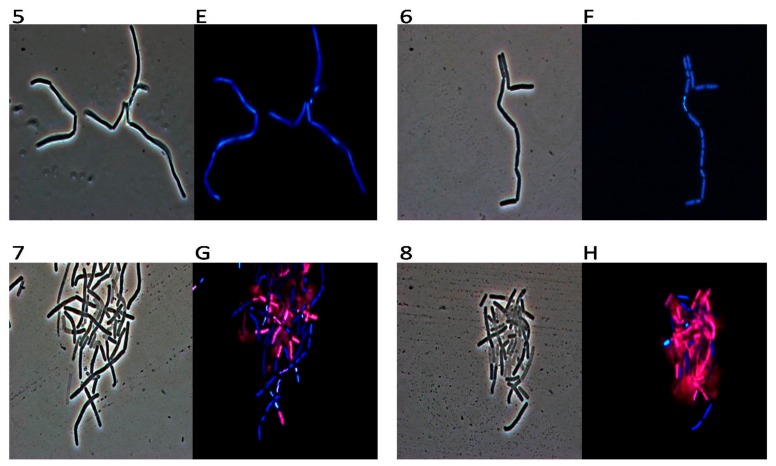
The panel shows *Bacillus cereus* bacterial cells observed in optical microscopy (numbers) and in fluorescence microscopy (letters). Untreated bacterial cells (**5**,**E**); cells treated with oil from MaH1 (**6**,**F**), cells treated with oil from MaH2 (**7**,**G**) and finally with oil from MaH3 (**8**,**H**).

In order to gain an insight into the possible mechanism of action of the active molecule present in different mint essential oils, we used fluorescence microscopy to study their effect on the selected bacterial strain. We have chosen *Bacillus cereus* for the following microscopy experiments. *Bacillus cereus* is a significant cause of toxin-induced food poisoning characterized by emesis and diarrhoea. Although a variety of foods have been implicated, including infant cereal, contaminated fried rice most frequently is associated with the emetic form of disease, which is caused by heat-resistant, preformed toxin, cereulide, or *B. cereus* spores that germinate when boiled rice is left unrefrigerated [17]. The organism typically is introduced by a projectile foreign body or open globe injury, producing a rapidly severe infection that frequently results in enucleation or poor visual outcome. A postsurgical endophthalmitis outbreak has been described, but *B. cereus* is an uncommon cause of postoperative endophthalmitis. Management is aggressive, including surgical intervention and parenteral, intravitreal and topical antimicrobial treatment. *B. cereus* is resistant uniformly to β-lactam antibiotics. An experiment was carried out to evaluate the interaction between the oils and the *B. cereus* strain using DAPI as DNA fluorescent stain and the fluorescent PI dye, that is only able to penetrate damaged membranes and is generally excluded from viable cells. Our results, obtained using 1% *v*/*v* of MpH1 essential oil, showed that all the treated cells emit red fluorescence light as a consequence of an impairment of membrane integrity (Figure 5(2,B)). The other oils have an intermediate amount of red and blue cells, very consistent with the antimicrobial activity measured by the inhibition halo and described by dose-response curves and MIC (Figure 5(3,C,4,D)). The only oil that does not seem to possess antimicrobial activity and does not allow the PI to enter inside the cells is MaH1, in fact, as we can see in Figure 6(6,F), bacterial cells appeared blue. As expected, fluorescence of untreated cells was blue because PI could not cross their intact plasma membranes (Figure 5(1,A) and Figure 6(5,E)).

**Figure 7:**
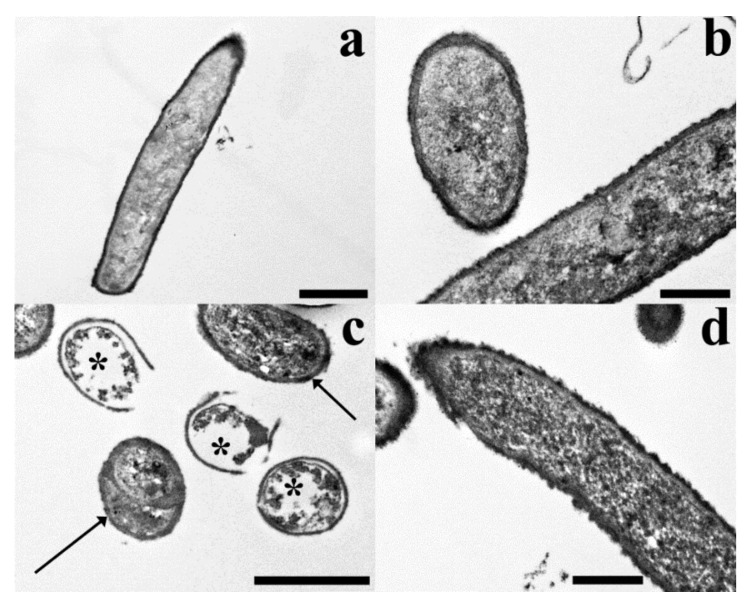
The figure shows TEM micrographs of untreated control (**a**,**b**) and Mph1 fraction-treated (**c**,**d**) *B. cereus* samples. **Untreated control samples**. (**a**) A longitudinal section of an untreated control bacterium with a quite electron dense protoplasm surrounded by an electron dense cell wall. (**b**) Cross and longitudinal sections of control bacteria where the dense texture of the cell walls is shown. **Mph1 fraction-treated samples**. (**c**) The micrograph shows both control-like (arrows) and changed bacteria (asterisks), the latter with very electron clear protoplasm surrounded by well visible cell walls. (**d**) A detail of a bacterial cell with a control-like appearance. Scale bars: 1 µm (**a**,**c**), 500 nm (**b**,**d**).

Our TEM observations showed that the untreated control cells had quite electron dense protoplasm surrounded by a very electron dense cell wall with a solid texture (Figure 7a,b). Treatments with mint extracts induced the appearance of changed bacteria with electron clear or even almost empty protoplasm delimited by still well visible, electron dense cell walls. Control-like bacteria are still present, along with altered cells, despite the treatments and even at the highest concentration (Figure 7b,c).

## 3. Discussion

The essential oils of *Mentha* x *piperita* L. var. Mitcham and *Menthaarvensis* var. *piperascens* were found to be particularly rich in oxygenated monoterpenes (Table 1). In the mint essential oil, these are surely the most important metabolites. Menthol, menthone and their isomers, menthyl esters and piperitone are the compounds responsible for the typical peppermint flavour[18]. Menthol, besides providing the classic minty note, also activates the cold-sensitive receptors in the oral cavity, producing a cooling effect. Menthofuran adds a sweet and hay-like minty odour.

The effect of heat stress on the chemical composition of the essential oils of *M. piperita* (Mp) and *M. arvensis* (Ma) is showed in Table 1. As we can see, the percentages of menthol seem to decrease for both the mint species at the increasing of the temperature, while on the contrary the percentages of both pulegone and menthyl acetate increase as temperature rises. Other components that vary strongly at the different temperatures are menthone and menthofuran, whose percentages are greatly affected by heat stress.

The antimicrobial activity and the MICs of the essential oils of the two mint species was also analysed; the tested oils showed a strong and dose-dependent antimicrobial activity, also directed against pathogenic strains resistant to antimicrobial substances [19].

Heat treatment significantly affects the antimicrobial activity of the oils. In particular, heat shock seems to greatly improve the antimicrobial activity of oils extracted from *M. arvensis* (MaH2 and MaH3). We successively tried to understand the mechanism underlying the toxicity against bacteria, with the use of fluorescence microscopy. Our data show that the possible target of the active molecule(s) extracted from *M. piperita* and *M. arvensis* is the bacterial membrane. In support of this hypothesis, membrane damage seems to be strongly related to the antimicrobial activity of the various oils. In order to confirm the fluorescence microscopy data, the samples treated with the MpH1 oil, the one showing the greatest antimicrobial activity, were observed by a transmission electron microscopy (TEM) analysis. Under TEM microscopy, the untreated control bacteria had a well-known typical appearance of quite electron dense protoplasm delimited by a solid cell wall. The changed appearance of the bacteria suggests that the protoplasm, under the toxic effect of the mintoil, loses most or even all of its content, giving an electron clear or even empty appearance to the cell. Nevertheless, the cell walls still featured a solid, electron dense structure. All this suggests that the oil induced the lysis of the protoplasm, whilst not damaging the ultrastructure of the cell wall. This is consistent with the hypothesis that the cell membrane is the target of the mint essential oil. In fact, it was already reported that monoterpenes and particularly menthol, act against both the gram-positive and the gram-negative bacteria damaging biomembranes, in particular perturbing the lipid fraction of microorganism plasma membrane, resulting in alterations of membrane permeability and in leakage of intracellular materials [20]. Furthermore, the oil might cross the cell membranes, penetrating into the interior of the cell and interacting with intracellular sites critical for antibacterial activity. Since it can be related to the physicochemical characteristics of the drugs (such as lipophilicity and water solubility), this effect seems to be dependent on lipid composition and net surface charge of microbial membranes.

Our results suggest that heat treatments strongly affect chemical composition and antibacterial activities of essential oils extracted from mint species. Since oil composition of this economically important genus is crucial for its exploitation, the growing conditions and the temperature should be optimized and monitored with attention, to obtain an oil with the desired characteristics.

## 4. Materials and Methods

### 4.1. Plant Material, Culture and Treatment

*Mentha* x *piperita* L. var. Mitcham and *Mentha arvensis* var. *piperascens* Malinv. ex L. H. Bailey were obtained from “Safiabad agricultural and natural resources research and education centre.”The phenologic stage of the collected plants was “bud formation” and the official gene bank where voucher specimens are deposited is the “Medicinal Plants and Drugs Research Institute Herbarium (MPH), Shahid Beheshti University of Tehran”.

**Figure 8:**
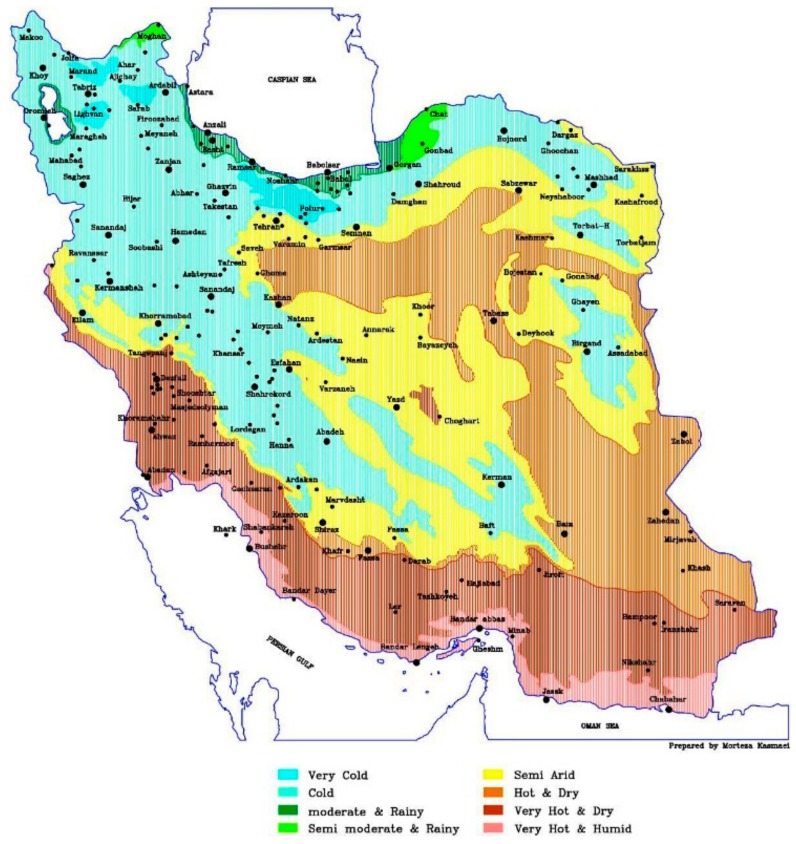
Different temperatures and humidity areas of Iran Moderate and rainy (H1) hot and dry (H2); very hot and dry (H3).

Ten cm of rhizomes in 21 cm pots (4 Lit) were planted and stored for 7 months in the greenhouse. The chemical and physical properties of the soil were: soil texture = loam, Sand = 38%, Silt = 38%, Clay = 24%, Ca = 7.7%, organic matter = 0.84%, Exchange Na = 1.48%, EC = 1.98 dS·m^−1^, pH = 7.8, available K = 151 mg·kg^−1^, available P = 14.1 mg·kg^−1^, total N = 0.09% for 4/5 plus leaf soil to improve the physical properties of soils for 1/5. In the field and in nature, the heat stress happened only in the third harvest (in summer in the hot area) so in this period in two-stages, we harvested the shoots. To choose the heat stress temperature to use, we analysed the variation of temperature in different areas of Iran, considering the average of 50 years meteorological data. Therefore, we individuated two different heat stress conditions (hot and extremely hot), to compare to the moderate climate. The hottest area is particularly important because around the city of Dezful, Khuzestan there is a big river for irrigation and the biomass of peppermint is higher than in other areas (more growth rate, more harvest per year and longer season growing); in Figure 8, the extent of hot areas in Iran are shown. Considering these data, we programmed the growth chamber (16/8 h brightness/darkness) and step by step we increased the temperature during the 20 days, from a normal cycle of temperature occurring in moderate climatic of Iran (14–32 °C) in H1, to warm cycle corresponding to hot areas of Iran (26–40 °C) in H2 and to the very hot cycle corresponding in very hot Iranian areas (27–45 °C) in H3. Temperature conditions for heat stress are showed in Table 3. Photosynthetic Active Radiation was 400 μmol m^2^ s^−1^(max light in growth chamber) and humidity was 70% during the treatment. Samples were planted in the growth chamber for 40 days. In the tenth day of treatment irrigated the plant by 80 cc/pot (of complete fertilizer 20-20-20 N-P-K, 2 g/1000 cc).

At each site, 50 sample plants were selected depending on the population size with a minimum distance of 100 m, then mixed for homogenization and used in three replicates for essential oil extractions.

### 4.2. Isolation of Essential Oils

The aerial parts of the studied samples were air-dried in the shade at the room temperature and then ground in a Waring blender. To isolate the essential oils, each sample (50 g in three replications) was extracted using hydro-distillation for 3 h, using a Clevenger-type apparatus based on the standard procedure described previously [20]. The essential oils were obtained with different yields (% *w*/*w* based on dry weight, see Figure 1) and were yellowish, with a pleasant smell. The oils were dried over anhydrous sodium sulphate and stored under N_2_ at +4 °C in the dark until tested and analysed.

The oils extracted from *Mentha piperita* were called MpH1, MpH2 and MpH3 because subjected to three different temperature ranges: H1 (14–32 °C), H2 (26–40 °C) and H3 (27–45 °C). The oils extracted from *Mentha arvensis* were called MaH1, MaH2 and MaH3 because subjected to the same temperature ranges of the other plant.

### 4.3. GC and GC-MS Analysis

Analytical gas chromatography was carried out on a Perkin-Elmer Sigma 115 gas chromatograph fitted with a HP-5 MS capillary column (30 m × 0.25 mm), 0.25 μm film thickness. Analysis was also run by using a fused silica HP Innowax polyethylenglycol capillary column (50 m × 0.20 mm), 0.20 μm thick film. Gas chromatography analysis has been performed as described in detail previously [21]. Identification of the compounds and components relative percentages was carried out as described in [21].

### 4.4. Bacterial Strains 

The antimicrobial activity was evaluated using gram positive strains: *Staphylococcus epidermidis*, *Staphylococcus aureus*, *Bacillus subtilis* and *Bacillus cereus*.

### 4.5. Antimicrobial Activity Assay

The presence of antimicrobial molecules in the essential oils was detected using the agar diffusion assay following the method of *Kirby-Bauer* with slight modifications [16]. Briefly, 5 μL of essential oil were placed on Muller Hinton agar plates, which were overlaid with approximately 8 mL of soft agar (0.7%) pre-mixed with 10 μL of *Staphylococcus epidermidis*, *Staphylococcus aureus*, *Bacillus subtilis and Bacillus cereus* strains grown for 24 h at 37 °C. Plates were incubated overnight at 37 °C and the anti-microbial activity was calculated as a measure of anti-microbial molecule production, according to Equation (1) [22,23]:

(1) $$A/\mathrm{mL}=\frac{\mathrm{Diameter}\;\mathrm{of}\;\mathrm{the}\;\mathrm{zone}\;\mathrm{of}\;\mathrm{clearance}\;\left(\mathrm{mm}\right)\;\times 1000}{\mathrm{Volume}\;\mathrm{taken}\;\mathrm{in}\;\mathrm{the}\;\mathrm{well}\;\left(\mu L\right)}$$

A single colony of every strainwas re-suspended in 5 mL of LB medium (Difco, Detroit, MI, USA) and incubated over-night at 37 °C. When the culture reached an OD_600_ of 1 unit, it was diluted 1:100 in 20 mM, pH 7.0 NaP buffer. Samples with a final volume of 500 μL were prepared; they contained bacterial cells for 1/25 of the final volume and essential oils (MpH1, MpH2, MpH3, MaH1, MaH2 and MaH3) at 0.005% 0.01% 0.2%, 1% *v*/*v* concentration and 20 mM pH 7.0 of NaP buffer up to final volume. Samples without essential oils were used as a control. After 2 h of incubation at 37 °C with stirring at 150 rpm serial dilutions (1:100, 1:1000) of all samples were prepared and then plated on LB-agar in Petri dishes that were finally incubated at 37 °C overnight. The following day, the surviving percent of bacterial cells was estimated by counting the number of colonies [24]. Each experiment was performed in triplicate and the reported result was an average of three independent experiments. (*p* value was < 0.05).

### 4.6. Determination of Minimal Inhibitory Concentration

Minimal inhibitory concentrations (MICs) of all essential oils against the different strains were determined according to the microdilution method established by the Clinical and Laboratory Standards Institute (CLSI). ~5 × 105 cfu/mL were added to 95 μL of cation-adjusted Mueller-Hinton broth (CAM-HB; Difco) supplemented or not with various concentration (0.005-0.01-0.2-1% *v*/*v*) of oils extracted from *Mentha* x *piperita* (MpH1, MpH2, MpH3)and *Menta arvensis* (MaH1, MaH2, MaH3) [25]. Growth was determined after 20 h incubation at 37 °C.

### 4.7. DAPI/PI Dual Staining and Fluorescence Microscopy Image Acquisition

For dual staining, 100μL of the bacterial culture of *Bacillus cereus* (bacteria were grown to mid-logarithmic phase) was incubated in the dark for 2 h at 37 °C in agitation in the presence or absence of oils (MpH1, MpH2, MpH3, MaH1, MaH2 and MaH3), a concentration of 1%. After the incubation, 10 μL of bacterial culture was mixed with DAPI solution (4′,6-diamidino-2-phenylindole dihydrochloride; Sigma Aldrich, Milan, Italy) (1 μg/mL DAPI final concentration) and PI (propidium iodide; Sigma Aldrich, Milan, Italy) 20 μg/mL. Samples were observed using an Olympus BX51 fluorescence microscope (Olympus, Tokyo, Japan) using a DAPI filter (excitation/emission: 358/461 nm). Standard acquisition times were 1000 ms for DAPI/PI dual staining. Images were captured using an Olympus DP70 digital camera [26].

### 4.8. TEM (Transmission Electron Microscopy)

Samples were fixed in 3% glutaraldehyde in phosphate buffer (pH 7.2–7.4) for 2 h at room temperature and post-fixed with buffered 1% OsO4 for 1.5 h at room temperature, dehydrated with ethanol and propylene oxide and embedded in Spurr’s epoxy medium [27]. At every step, bacterial suspension was centrifuged at 2000 rpm for 5 min so to obtain a pellet and the supernatant was substitutes with the following agent. After a 2 day-polymerization in an oven at 70 °C, resin blocks were cut into ultra-thin (50 nm thick) sections, which were mounted on 300-mesh copper grids, then stained with Uranyl Acetate Replacement stain UAR (Electron Microscopy Sciences) and lead citrate and observed with a Philips EM 208S TEM. Electron microscopy observations were only made on MpH1 fraction-treated *B. cereus* samples.

### 4.9. Statistical Analysis

Two factors (two mint species and three heat regimes) were studied in factorial by three replications in a completely randomized design (CRD). All the treatments were performed for 40 days (simulation of native place and day).

## Figures and Tables

**Table 1:** Chemical composition of essential oils of the two mint species (Mp = *M. piperita*, Ma = *M. arvensis*, H1, H2 and H3 are the three tested temperatures). Each value is the mean ± SD of three independent measurements.

LRI ^a^	LRI ^b^	Component	MpH1	MpH2	MpH3	MaH1	MaH2	MaH3
		Monoterpene hydrocarbons	4.3	3.6	6.7	3.9	3.6	5.0
938	1075	α-Pinene	0.7 ± 0.01	0.6 ± 0.01	0.7 ± 0.02		0.4 ± 0.01	0.6 ± 0.01
973	1132	Sabinene	0.3 ± 0.02				0.4 ± 0.01	
975		*p*-2-menthene	0.1 ± 0.01					
978	1118	β-Pinene	0.7 ± 0.02	0.6 ± 0.02	0.7 ± 0.03		0.7 ± 0.02	0.7 ± 0.01
987		*p*-3-menthene	0.4 ± 0.01	0.6 ± 0.01	2.7 ± 0.09	3.9 ± 0.06	0.6 ± 0.03	1.1 ± 0.06
993	1174	Myrcene	0.1 ± 0.01	0.1 ± 0.01				
1029	1218	β-Phellandrene	0.3 ± 0.01	0.3 ± 0.01	0.3 ± 0.01			0.4 ± 0.01
1030	1203	Limonene	1.7 ± 0.03	1.4 ± 0.04	2.3 ± 0.07		1.5 ± 0.05	2.2 ± 0.04
		**Oxygenated monoterpenes**	**92.0**	**94.8**	**90.6**	**88.3**	**92.5**	**90.3**
1034	1213	1,8-Cineole	0.3 ± 0.01	0.3 ± 0.01	0.3 ± 0.01		3.7 ± 0.09	1.8 ± 0.01
1063	1550	(Z)-Sabinene hydrate					0.4 ± 0.01	
1150	1475	Menthone	14.5 ± 0.09	7.6 ± 0.08	11.9 ± 0.13	13.0 ± 0.09	15.9 ± 0.06	9.5 ± 0.04
1164	1460	Menthofuran	7.8 ± 0.12	33.9 ± 0.14	12.9 ± 0.18	35.0 ± 0.13	25.4 ± 0.08	34.0 ± 0.19
1165	1570	Neomenthol	1.6 ± 0.07		6.8 ± 0.08	1.3 ± 0.05		
1173	1626	Menthol	56.6 ± 0.34	38.5 ± 0.21	27.5 ± 0.16	24.3 ± 0.16	12.2 ± 0.05	5.6 ± 0.07
1234	1662	Pulegone	5.6 ± 0.16	7.8 ± 0.19	10.9 ± 0.11	14.7 ± 0.09	24.3 ± 0.12	28.1v0.11
1291	1541	Menthyl acetate	2.1 ± 0.11	4.5 ± 0.09	15.4 ± 0.14		4.8 ± 0.07	6.1 ± 0.03
1343	1748	Piperitone	0.5 ± 0.03	0.2 ± 0.01				
1366	1983	Piperitenone oxide	1.9 ± 0.04	1.7 ± 0.03	3.7 ± 0.07		4.3 ± 0.03	3.9 ± 0.02
1579	2008	Isomenthone	1.1 ± 0.01	0.3 ± 0.01	1.2 ± 0.02		1.5 ± 0.02	1.3 ± 0.01
		**Sesquiterpene hydrocarbons**	**0.6**	**0.6**	**0.5**	**6.4**	**1.6**	**2.2**
1418	1612	β-Caryophyllene	0.3 ± 0.01	0.4 ± 0.02	0.5±0.02	0.7±0.03	0.9 ± 0.04	1.2 ± 0.04
1458		t-β-farnesene	0.1 ± 0.01	0.1 ± 0.01				0.2 ± 0.01
1463	1667	Alloaromadendrene	0.2 ± 0.01			2.6 ± 0.09	0.4 ± 0.01	0.3 ± 0.01
1477	1726	Germacrene D	-	0.1±0.01		3.1 ± 0.08	0.3 ± 0.01	0.5 ± 0.01
		**Oxygenated sesquiterpenes**	**0.2**		**0.3**		**0.3**	**0.3**
1580	2150	Caryophyllene oxide	0.2 ± 0.01		0.3 ± 0.01		0.3 ± 0.01	0.3 ± 0.02
		**Others**	**2.3**	**0.8**	**1.8**		**1.8**	**2.0**
1195	1655	Methylchavicol	0.5 ± 0.01	0.2 ± 0.01	0.4 ± 0.01		0.4 ± 0.01	0.2 ± 0.01
1446	2345	Mint-furanone I	1.8 ± 0.05	0.6 ± 0.02	1.4 ± 0.04		1.4 ± 0.03	1.8 ± 0.02
		**Total**	**99.4**	**99.8**	**99.9**	**98.6**	**99.8**	**99.8**

^a^ Ki: retention index on a HP-5 MS column. ^b^ Ki: retention index on an Innowax column.

**Table 2:** Minimal inhibitory concentration (MIC) of the essential oils from *Mentha* x *piperita* and *Mentha arvensis*.

	MIC (mg/mL) *					
Bacterial Strains	MpH1	MpH2	MpH3	MaH1	MaH2	MaH3
*S. epidermidis*	2	8	18.5	10	10	10
*S. aureus*	10	12	20	10	10	10
*B. subtilis*	0.1	2.5	0.06	10	10	0.1
*B. cereus*	0.08	8	10	10	15	2

* MIC values shown in the table are the highest concentrations obtained after three independent experiments.

**Table 3:** Temperature conditions for heat stress. H1: normal, H2: heat stress, H3: extreme heat stress.

T3	T2	T1	Light	Hours in a Day
31	28	18	dark	24–4
27	26	14	dark	4–8
37	33	24	light	8–13
45	40	32	light	13–19
38	33	25	light	19–24
